# Evolving Nutritional Needs in Cystic Fibrosis

**DOI:** 10.3390/life13071431

**Published:** 2023-06-22

**Authors:** Theresa Frantzen, Sara Barsky, Geralyn LaVecchia, Michelle Marowitz, Janice Wang

**Affiliations:** 1 Division of Pulmonary, Critical Care and Sleep Medicine, Donald and Barbara Zucker School of Medicine at Hofstra/Northwell, New Hyde Park, New York, NY 11042, USA; glavecch@northwell.edu (G.L.); jwang@northwell.edu (J.W.); 2 Division of Pediatric Pulmonology, The Steven and Alexandra Cohen Children’s Medical Center, Donald and Barbara Zucker School of Medicine at Hofstra/Northwell, Lake Success, New York, NY 11042, USA; mmarowitz@northwell.edu

**Keywords:** cystic fibrosis, nutrition, highly effective modulator therapy, body mass index, pancreatic insufficiency, cystic fibrosis-related diabetes, cystic fibrosis pregnancy

## Abstract

The course of cystic fibrosis (CF) as a nutritional illness is diverging since the introduction of highly effective modulator therapy, leading to more heterogeneous phenotypes of the disease despite CF genetic mutations that portend worse prognosis. This may become more evident as we follow the pediatric CF population into adulthood as some highly effective modulator therapies (HEMT) are approved for those as young as 1 year old. This review will outline the current research and knowledge available in the evolving nutritional health of people with CF as it relates to the impact of HEMT on anthropometrics, body composition, and energy expenditure, exocrine and endocrine pancreatic insufficiencies (the latter resulting in CF-related diabetes), vitamin and mineral deficiencies, and nutritional health in CF as it relates to pregnancy and lung transplantation.

## 1. Background

Nutritional manifestations of cystic fibrosis (CF) are a principal focus of interest in the evolving landscape of CF care. People with CF (PwCF) have a higher prevalence of malnourishment compared to the general population as a consequence of its underlying pathophysiology in multiple organ systems, particularly the pancreas. Historically, CF has been linked to nutritional malabsorption causing failure to thrive and death in children. In 1938, autopsy findings from pediatric patients with CF were reported by Dr. Dorothy Andersen to show extensive pancreatic ductal obstruction and fibrosis and mortality due to severe malnutrition [1]. About 90% of individuals suffer from exocrine pancreatic insufficiency (PI) which causes malabsorption of lipids and fat-soluble vitamins, weight loss, diarrhea, risk for zinc deficiency, and stunted growth in children. CF is, in fact, also strongly linked to high-energy and nutrient needs due to its respiratory manifestations, increased metabolic rate and chronic inflammatory state [2]. Additionally, CF-related diabetes, anorexia, dysphagia, bone disease, and chronic medication usually result in nutritional concerns as well. CF nutritional care encompasses nutrition education, counseling, specialized diets to maintain body mass index (BMI) goals, and respiratory and overall health. Nutritional and lung health are strongly correlated; PwCF who have optimized nutrition and BMI have reduced risk of infection, morbidity and mortality [2]. 

Earlier CF therapies targeted nutritional malabsorption and respiratory clearance and infections with the use of pancreatic enzyme replacement therapy (PERT), inhaled antibiotics, and mucolytic treatments. Fast forward to the era of cystic fibrosis transmembrane conductance regulator (CFTR) modulators, also referred to as highly effective modulator therapy (HEMT), and the majority of PwCF are now benefiting from these treatments that aim to restore CFTR activity at the cellular level. Modulators correct the defective CFTR protein and improve chloride ion transport in epithelial cells of multiple organs, thereby improving organ function and delaying organ injury [3]. For PwCF who qualify for modulator therapy, it is an imperative standard of care. Commercially available CFTR modulators include ivacaftor (IVA), lumacaftor/ivacaftor (LUM/IVA), and tezacaftor/ivacaftor (TEZ/IVA). These drugs have demonstrated various effects on nutrition, weight, and body mass index (BMI) over the last decade. For example, IVA had positive effects on weight gain, yet TEZ/IVA and LUM/IVA produced either no significant changes or mixed effects in weight and BMI [4]. The latest approved modulator therapy is known as elexacaftor/tezacaftor/ivacaftor (ETI), tradename Trikafta^®^. Trikafta is a triple drug modulator therapy, and was approved in 2019 by the United States Food and Drug Administration (FDA) for PwCF ages 12 and older. In contrast to prior modulators’ variable effects on anthropometric markers, ETI has shown significant improvements in weight and BMI. In 2021, ETI approval expanded to those 6 years of age and older with at least one F508del mutation and has made dramatic improvements in lung function as measured by forced expiratory volume in one second (FEV1), reduced pulmonary exacerbations and increase in weight and BMI [4,5]. About 90% of the CF population in the United States carries at least one F508del mutation and are therefore eligible for or receiving treatment with ETI [6]. In 2023, ETI became FDA approved for children with CF ages 2 to 5 years who have at least one copy of the F508del mutation or certain mutations that are responsive [7]. 

Following ETI use, long-term oxygen, non-invasive ventilation, and enteral nutrition needs have reduced by up to 50% in PwCF, as well as reductions in hospitalizations and lung transplantations [8]. The PROMISE study (A Prospective Study to Evaluate Biological and Clinical Effects of Significantly Corrected CFTR Function) is a real-world observational study evaluating the effectiveness of ETI across multiple clinical outcomes for up to 4.5 years (NCT04038047) [9]. One aspect to be explored is to determine whether fecal elastase values, which are diagnostic of PI (i.e., fecal elastase ≤ 200 µg/g), may increase in younger PwCF while on HEMT; this would support the hypothesis that ETI may safeguard against or normalize exocrine pancreatic function to some degree [10]. These positive changes were already observed in case studies of older children with PI who began IVA, and thus ETI may provide similar benefits [11,12,13]. 

The clinical changes seen thus far with ETI mean a promising future for how HEMT will affect nutrition in PwCF. Emphasis also remains on the continued nutritional care of patients who are ineligible for HEMT or remain underweight or malnourished despite HEMT use, such as those with advanced CF lung disease. This review covers the impact of HEMT on anthropometrics, body composition, energy expenditure, exocrine PI, CF-related diabetes, vitamin and mineral deficiencies, and nutritional health in CF as it relates to pregnancy and lung transplantation. 

## 2. Anthropometric Parameters

Anthropometry, the study of body measurements, has been invaluable in our understanding of nutrition and overall growth, and medical and physical health in PwCF. Anthropometric outcomes commonly evaluated in clinical CF care and investigational studies are BMI percentiles and absolute value for pediatric and adult CF patients, respectively. Values that are associated with optimal lung function for pediatric patients include 50th percentile or above in weight-for-length for individuals up to age 2, greater than 50th percentile for BMI for ages 2–20, and for adults, a BMI of at least 22 for females and 23 for males. While weight gain is helpful in order to depict overarching anthropometric changes, significant detections in BMI are more pertinent due to the established correlation between BMI and lung function. As previously mentioned, ETI improves BMI status in PwCF and potentially PI, enabling BMI milestones to be achieved sooner and more easily with a probable decrease in caloric supplementation. These improvements were observed in people carrying at least one copy of the F508del mutation, across multiple published studies of varying duration; see Table 1.

While there are fewer studies assessing anthropometrics in the pediatric CF population, especially for ages 6–11, weight and BMI outcomes parallel that of adult trials [14]. ETI is associated with substantial weight gain as seen in underweight individuals with eventual normalization of BMI, although in PwCF with a normal BMI, continued weight gain may potentially lead to increased prevalence of obesity [15]. It is unclear whether ETI-associated weight gain is compensatory or excessive, especially considering this weight gain may be a response to years of malnutrition. Burgel et al. suggests that the degree of weight gain may be attributed to previously achieved weight gain from prior CFTR modulator use such as that observed in F508del homozygous individuals treated with LUM/IVA [8]. However, Graeber et al. showed weight gain after ETI to be equivalent or greater in patients treated with a modulator at baseline compared to no prior modulator [16]. Improved weight following HEMT may also be a downstream effect of a reduced hyperinflammatory state and reduced energy expenditure. In most PwCF, energy expenditure is higher than that of those without CF due to a systemically higher inflammatory state, particularly from the respiratory system where there is chronic bacterial colonization and airway inflammation. Evidence supports HEMT-associated reduction in such inflammation which can conserve energy and body weight [17].

Weight-related outcomes for PwCF with pancreatic sufficiency and/or CF-related diabetes (CFRD) are inconclusive. Pancreatic-sufficient patients did not have any significant weight change following ETI therapy across studies, likely because their baseline weight and BMI were higher compared to those with PI. In addition, individuals with CFRD had mixed results with weight gain. Scully et al. showed that in a CFRD cohort, there was an overall statistically significant increase in body weight by 5.6 kg (*p* < 0.045), but a non-statistically significant increase in BMI of 1.3 kg/m^2^, likely because a larger weight increase is needed to detect a change in BMI [18]. Petersen et al. found a significant annualized BMI gain of 1.47 kg/m^2^ per year based over a 20-month period in patients with CFRD [19].

While significant changes in weight and BMI have been seen with ETI, it is vital to continue to vigorously trend the anthropometric effects of HEMT in PwCF. The anticipated “Strength and Muscle Related Outcomes for Nutrition and Lung Function in CF” (STRONG) trial is a prospective observational trial that will be studying markers of anthropometrics, body composition, sarcopenia and frailty and compare them to dual-energy X-ray absorptiometry (DXA) output in patients of 18 years and older with CF (NCT05639556). The data gathered will also be analyzed for psychosocial and clinical outcomes in the context of varying lung function severity [15]. Long-term observations will help us to further understand the effect of HEMT on nutrition and elucidate questions such as whether weight gain plateaus, signaling a metabolic equilibrium, or maintains a rising trajectory, posing a risk for obesity.

**Table 1:** Effect of elexacaftor/tezacaftor/ivacaftor (ETI) on weight and body mass index outcomes in individuals who possess at least one F508del mutation. In the referenced observational studies, all participants were initiated on ETI, and changes in BMI/weight are noted. For interventional trials, ETI was compared to control (either placebo or tezacaftor/ivacaftor). * Anthropometrics based on Wang et al. (2012) Handbook [20]. BMI = body mass index; RCT = randomized controlled trial, CFTR = cystic fibrosis transmembrane conductance regulator; ETI = elexacaftor/tezacaftor/ivacaftor; LS = least squares mean; CFRD = cystic fibrosis related diabetes.

Study	Study Design and Duration	Age Range	F508del Mutation	The Effect of ETI on Weight/BMI
Burgel et al. (2021) [8]	Prospective observational study; 8 months	12+	Homozygous, Heterozygous	All participants were initiated on ETI and had an average weight gain of + 4.2 kg (*p* < 0.00001) No significance or mentions of BMI
Carnovale et al. (2022) [5]	Retrospective observational study; 48 weeks	12+	Homozygous, Heterozygous	Mean (95% CI) absolute increase in BMI after various treatment intervals:
				- 4 weeks: 0.66 (0.37, 0.95; *p* < 0.0001) - 12 weeks: 1.57 (1.19, 1.94; *p* < 0.0001) - 24 weeks: 2.02 (1.56, 2.48; *p* < 0.0001) - 48 weeks: 2.08 (1.63, 2.52; *p* < 0.0001)
Fajac et al. (2022) [21]	Randomized controlled trial; 24 weeks	12+	Homozygous, Heterozygous	For LS mean absolute change, ETI group had significant increases in BMI compared to controls:
				- F508del heterozygous: 13.2 (9.8, 16.6) - F508del homozygous: 7.5 (1.6, 13.3)
Graeber et al. (2021) [16]	Prospective observational study; 16 weeks	12+	Homozygous, Heterozygous	All participants started ETI and had the following changes in BMI:
				- Heterozygous: +1.1 (0.4–1.9), *p* < 0.001 - Homozygous with tezacaftor use at baseline; +1.2 (0.5–1.5), *p* < 0.001 - Homozygous without CFTR modulator at baseline; +0.3 (0.0–1.3), *p* < 0.01
Heijerman et al. (2019) [22]	RCT, double- blind, active controlled; 4 weeks	12+	Homozygous	Weight status/BMI in ETI compared to tezacaftor/ivacaftor (+0.60 kg/m^2^, 95% CI 0.41–0.79; nominal * *p* < 0.0001)
Middleton et al. (2019) [23]	RCT, double-blind, placebo-controlled; 24 weeks	12+	Heterozygous	Compared to placebo, participants on ETI had the following changes: BMI > 20 y: +1.04 kg/m^2^ (*p* < 0.001)—BMI z-score < 20 y: +0.3 (95% CI 0.17–0.43)
Nichols et al. (2022) [24]	Prospective, observational study; 6 months	11+	Homozygous, Heterozygous	After 6 months of ETI treatment for all participants: +1.24 kg/m^2^ for BMI and +0.3 for BMI-z-score
O’Shea et al. (2020) [25]	Observational Study; 7 months	Adults, age not defined	Homozygous, Heterozygous	All participants had an average increase in BMI by 1.5 kg/m^2^ (*p* < 0.00001)
Petersen et al. (2021) [15]	Retrospective study; 14 months	20+	Homozygous, Heterozygous	All patients were initiated on ETI and significant changes in BMI were detected in all genotypes with a mean difference of +1.65 kg/m^2^:
				- Highly significant annualized difference in BMI trajectory = 1.47 kg/m^2^/yr (95% CI: 1.08 to 1.87, *p* < 0.0001) - Highly significant changes (*p* < 0.0001) in annualized BMI trajectory for subgroups: CFRD, pancreatic insufficiency, prior modulator use, no prior modulator use, heterozygous, and homozygous; no change in BMI status for pancreatic sufficient
Scully et al. (2021) [18]	Prospective cohort; 10 months	18+	Homozygous, Heterozygous	No significance in change in BMI after ETI therapy for total cohort:
				- Change in BMI was greatest in CFRD subgroup +1.3 kg/m^2^ (*p* = 0.082). - Significant increase in body weight by 5.6 kg in CFRD subgroup (*p* < 0.045)
Sutharsan et al. (2021) [26]	RCT, double-blind, active- controlled; 24 weeks	12+	Homozygous	BMI increase for ETI compared to tezacaftor/ivacaftor:
				- For ≥20 y: [95% CI: 1.44 kg/m^2^ (1.07–1.82)] - For <20 y: BMI z-scores [95% CI: 0.51 (0.20–0.82)]
Zemanick et al. (2021) [14]	Non-randomized controlled trial; 24 weeks	6–11	Homozygous, Heterozygous	All pediatric patients were initiated on ETI and had the following significant z-score changes:
				- Weight-for-age z-score: +0.25 [0.16–0.33, *p* < 0.001] - BMI-for-age z-score: +0.37 [0.26–0.48, *p* < 0.001]

### Energy Expenditure and Body Composition 

More research is needed to understand the impact of HEMT on body composition, including fat mass and fat-free mass, energy expenditure, and dietary needs. Consumption of a fat-containing food with HEMT is imperative for absorption. Individuals with PI must also take PERT in conjunction with a fat-containing food and the medication. This factor may lead to an overall increase in caloric intake compared to pre-HEMT initiation, albeit LUM/IVA and TEZ/IVA did not result in significantly comparable weight gain compared to ETI [4]. Thus, the observed increased weight and fat mass may be attributed to the physiological effect of the drug as opposed to changes in caloric intake.

Body composition has become a key determinant of clinical outcomes in CF. Fat-free mass is positively correlated with lung health and overall outcomes while the opposite relationship can be said for fat mass as well [26]. With significant increases in weight and BMI across studies for those with CF and PI, understanding the way in which this weight is being deposited in the body has been a topic of investigation. Prior studies on IVA found an increase in fat mass and either an unchanged or increased effect on fat-free mass [27]. Gur et al. (2022) showed promising results for 18 individuals with CF taking ETI regarding the changes in body composition, bone mineral density (BMD), and exercise capacity. An increased weight of 2.5 kg (*p* = 0.05) and BMI of 0.9 kg/m^2^ (*p* = 0.05) was observed. Hip and spine BMD increased significantly in the treatment group; (0.73 ± 0.098 to 0.81 ± 0.12 g/cm^2^ for hip [*p* = 0.017]; and 0.76 ± 0.14 to 0.82 ± 0.14 g/cm^2^ for spine [*p* = 0.025]) compared to no difference in hip or spine BMD for the control group [28]. These results provide promising information for how ETI may change the progression of CF bone diseases. Additionally, there was a significant increase in lean body mass (*p* = 0.017) and a non-statistically significant change in fat mass percentage (*p* = 0.069) [26]. Six-minute walk testing used to assess endurance and aerobic capacity also significantly improved by a distance of approximately 50 additional meters (*p* = 0.046) [26]. 

Gur et al. (2022) suggests some mechanisms that may be responsible for the changes above, including reduced resting energy expenditure, increased fat absorption and decreased gut inflammation. Increased lean body mass is associated with reduced inflammation and improved lung function and CF disease severity. This is early evidence of how ETI is truly changing body composition and physiology [29]. Extended observations may reveal the potential for increased physical activity and weight gain through muscle mass, underscoring the need for multidisciplinary coordination of care between a dietitian and physical therapist to optimize the interplay between nutritional and physical health. While smaller case series have yet to demonstrate improved physical activity with muscle mass following initiation of ETI, continued research of longer duration may reveal more promising data [30]. 

## 3. Comorbidities with Nutritional Implications

CF, although primarily a lung disease, also causes a variety of gastrointestinal (GI) manifestations which are important for diagnosis, prognosis, and quality of life [31]. Common comorbidities include exocrine and endocrine PI, acute pancreatitis, gastroesophageal reflux disease, small-intestinal bacterial overgrowth, distal intestinal obstructive syndrome, and CF liver disease. GI-related symptom burden is high and includes abdominal pain, bloating, abnormal stools, constipation, and heartburn, found in as many as 80% of PwCF [32]. The nutritional implications of GI manifestations in CF are very influential in growth and development in children and malnutrition in all PwCF. Here, we review PI, vitamin and mineral deficiencies, CF-related diabetes, CF-related liver disease, and kidney stone disease in CF.

### 3.1. Exocrine Pancreatic Insufficiency

Exocrine pancreatic insufficiency (PI) is the most common GI complication of CF. The CFTR mutation contributes to impaired bicarbonate secretion, as well as obstruction of the pancreatic ducts by mucus, leading to fibrotic damage of the pancreas.

This damage causes inadequate pancreatic enzyme production, specifically of lipase, protease and amylase, which are important for digestion, absorption, and nutrient utilization of fats, proteins and carbohydrates, respectively. A fecal elastase-1 test measures the amount of elastase, which when low in level (fecal elastase ≤ 200 µg/g) is diagnostic of PI. Malabsorption due to PI can cause significant nutritional consequences of weight loss, malnutrition, and deficiencies in fat-soluble vitamins A, D, E, and K. Symptoms of PI include steatorrhea, abdominal bloating and distention. Most people with CF and PI will require pancreatic enzyme replacement therapy (PERT) to aid with nutrient utilization and prevent adverse nutritional outcomes and manage PI symptoms. Acid-suppressing medications including histamine H2 antagonists and proton pump inhibitors are also often prescribed to enhance the efficacy of PERT, as they work more effectively in a basic pH. Potential side effects of PERT may include abdominal cramps, nausea, vomiting, constipation, diarrhea, bloating and, in people taking high doses of enzymes, fibrosing colonopathy. It has been suggested that fibrosing colonopathy could be due to the presence of methacrylic acid copolymer in the capsule and not due to the high doses of enzymes [33]. Since the discontinuation of the use of the polymer there have been no reports of fibrosing colonopathy [34]. Polypharmacy is an enormous lifelong burden in majority of PwCF. A call for efforts to simplify or reduce medical burden was conveyed as one of CF patients’ top priority. PERT is no exception as it needs to be taken with food several times per day in numerous pill counts.

As mentioned previously, the PROMISE study aims to follow the real-world, long-term impact of ETI on GI comorbidities; one hypothesis is that it may delay the onset of PI or restore some exocrine function of the pancreas to the point of feasibly reducing PERT dosing [10,35]. Anecdotally, since the implementation of HEMT therapy, CF providers are recognizing the potential opportunity to reduce and/or withdraw PERT and acid-suppressing medications, but there is not enough evidence at this time to support this practice [36]. It is essential to continue to monitor for PI-related signs and symptoms and reassess PERT dosing at every clinic encounter.

### 3.2. Vitamins and Mineral Supplementation 

Deficiencies in vitamins and minerals are particularly common in PwCF and PI, although other contributing factors include uncontrolled fat malabsorption, poor adherence to PERT, and comorbidities such as short bowel syndrome and CF-related liver disease. Regular monitoring of fat-soluble vitamins and minerals annually and following supplement dosing changes are recommended. See Table 2.

#### 3.2.1. Vitamin A

Vitamin A is important for embryonic development and healthy bones, vision, and immune system. Vitamin A also has anti-oxidant properties that are protective in respiratory epithelial cells and overall cardiovascular health [37]. In CF, normal levels of vitamin A are linked to overall better lung function. Vitamin A can be consumed in two forms: preformed vitamin A (retinol, retinyl esters) and provitamin A carotenoids, which primarily include beta-carotene, alpha-carotene, and beta-cryptoxanthin in the human diet. Preformed vitamin A is found in foods such as dairy, liver, eggs, and fortified foods such as breakfast cereals. Provitamin A is found in plant-based foods (e.g., fruits, vegetables). Beta-carotene conversion into retinol occurs during states of deficiency and high oxidative stress [38]. Current recommendations for Vitamin A level range from 450–3000 mcg retinol equivalents per day; however, recommendations are not based on clinical trials [38,39]. Vitamin A deficiency is becoming rare in PwCF, although annual monitoring is still suggested based on its risk factor associated with PI. Elevated retinol serum is another clinically relevant concern related to vitamin A level and has been associated with liver and bone disease and increased intracranial pressure in the general population [37].

#### 3.2.2. Vitamin D

Vitamin D is important for normal bone mineralization and overall bone health by regulating calcium and phosphorus levels [40]. Compared to the general population, PwCF are at a greater risk of developing osteopenia and increased bone fractures. Causes for vitamin D deficiency include PI and decreased internal absorption, inadequate dietary intake or production via sunlight exposure, glucocorticoid use, and reduced fat stores. Vitamin D forms are ergocalciferol and cholecalciferol (Vitamin D3), the latter being the recommended form for supplementation. The primary source of ergocalciferol is plants and can be manufactured synthetically, whereas cholecalciferol is synthesized in the body from 7-dehydrocholesterol after exposure to the sun and is more effective in increasing vitamin D levels in the bloodstream longer than ergocalciferol [41]. The recommended daily goal for vitamin D ranges between 800–2000 IU daily and is the same recommended dose as the general population. Vitamin D should be monitored annually at the end of the winter season to assess the effects of limited sun exposure and to adjust supplemental doses as needed [41]. 

#### 3.2.3. Vitamin E

Vitamin E plays a role in protecting the body against harmful oxidative damage. Normal vitamin E levels range from 23 μmol/L to 46 μmol/L, and plasma levels over 80 μmol/L are considered excessive. There are eight forms of vitamin E, but alpha-tocopherol is the only form that meets human requirements. Foods rich in vitamin E include nuts, seeds, and oils. Vitamin E is best measured using a vitamin E to total cholesterol ratio, as vitamin E circulates in the blood bound to lipoproteins. Thus, elevated total cholesterol would falsely lower vitamin E levels unless both values are interpreted using a ratio. Vitamin E/lipid ratio (mg/g) is calculated based on the following formula: vitamin E (mg/L) ÷ [Total Cholesterol (g/L) + Triglycerides (g/L)]. Normal range for this ratio is 1.4–5.71 mg/g, while deficiency is indicated by a ratio < 0.8 mg/g [42]. Vitamin E deficiency is rare, but can have detrimental effects, such as peripheral neuropathy, myopathy, and pigmented retinopathy. The current recommended dosages of vitamin E range from 100 IU to 400 IU per day for PwCF [43]. The specific mechanism of action for most of its effect is still unknown. Elevated vitamin E is common in patients who are post-solid organ transplant and is associated with risk for major bleeding. 

#### 3.2.4. Vitamin K

Vitamin K is essential for blood clotting, bone formation, and cellular growth and is protective against free-radical induced cellular damage. Dietary sources are dark leafy green vegetables, such as kale, broccoli, and spinach. Causes of vitamin K deficiency are PI and frequent antibiotic use (causing changes in intestinal flora), commonly observed in PwCF. Vitamin K monitoring is assessed using prothrombin time and intake should be monitored carefully in patients on anticoagulants. Goal daily level recommended ranges; the Cystic Fibrosis Foundation (CFF) consensus guidelines recommend a vitamin K dose of 0.3–0.5 mg daily for children and 2.5–10 mg weekly for adults [44]. Additional vitamin K supplementation is commonly prescribed short- and long-term for patients with history of hemoptysis and concern for recurrence, but there are no clinical trials assessing the efficacy and dosing of vitamin K in the prevention of hemoptysis. In a retrospective observational study, vitamin K was prescribed to 38 adult CF patients in the setting of hemoptysis during an acute pulmonary exacerbation. Average vitamin K dose was 10 mg and two patients developed a thromboembolism. The benefit of vitamin K in CF patients with hemoptysis remains unclear [45].

With HEMT, vitamin deficiencies may be lessening, leading to overdosing in supplementation. Sommerburg et al. (2021) studied the effect of LUM/IVA on concentrations of lipid-soluble vitamins in CF patients. The study showed that vitamin D levels increased slightly after one year although this was not significant. Retinol levels increased following two years of LUM/IVA treatment; vitamin E levels decreased after one and two years on LUM/IVA [46]. Individuals might be prescribed modular formula vitamins or may need adjustments in their vitamin regimen. Modulator formula vitamins are lower in preformed vitamin A (retinol) and higher in pro-form vitamin A (beta carotene). Since most vitamin recommendations are not based on clinical trials, future research is needed to determine proper vitamin supplementation, especially in patients on HEMT.

**Table 2:** Vitamins and minerals of interest in cystic fibrosis. The above-listed vitamins and minerals are commonly found to be deficient as a result of poor absorption and pancreatic insufficiency. Daily goals of these vitamins and minerals are general recommendations from expert consensus rather than evidence-based practice and may not be CF-specific [47,48]. Adapted with permission from Sankararaman et al, 2022, John Wiley and Sons.

Vitamin/ Mineral	Dietary Sources	Important Roles	CF-Specific Relevance	Daily Goal Intake
Vitamin A	Dairy, liver, eggs, fortified foods (e.g., cereals)	Embryonic development, bone health, vision, Immune system	Deficiency may lead to poor eye, skin, and respiratory health	Infants: 1500 IU Toddlers: 5000 IU 4–8 years: 5000–10,000 IU Adults: 10,000 IU
Vitamin D	Salmon, tuna, milk, eggs, fortified cereal, cheese	Bone health	Higher prevalence of osteoporosis in CF versus general population	Infants: 400–500 IU 1–10 years: 800–1000 IU Adults: 800–2000 IU
Vitamin E	Nuts, seeds, oils	Antioxidant, protection of cells from oxidative stress	Protective of cell membranes, blood cells and nerves	Infants: 40–50 IU Toddler: 80–150 IU 4–8 years: 100–200 IU Adults: 200–400 IU
Vitamin K	Leafy greens, liver, soybean, canola oil	Coagulable function, bone health	Essential for blood clotting, and strong bones	Infants/Toddlers: 0.3–0.5 mg Adults: 2.5–10 mg/weekly
Iron (Fe)	Red meat	Oxygen-carrying capacity		Infants: 0.27 mg Toddlers: 7 mg–11 mg 4–8 years: 10 mg Adults: 8 mg
Zinc	Red meat, chicken, fortified cereals, beans, nuts, whole grains	Immune system, wound healing		Infants: 1 mg/kg/day 4–8 years old: 15 mg/day Adults: 25 mg/day
Magnesium	Whole grains, nuts, legumes, avocado	Muscle and nerve function, bone health		Currently no formal guidelines for treatment of magnesium in CF
Calcium	Dairy, Milk	Bone health		Same recommendation as the general population Infants: 200 mg Toddlers: 700 mg 4–8 years: 1000 mg Adults 1000–1300 mg

### 3.3. Endocrine Pancreatic Insufficiency—Cystic Fibrosis-Related Diabetes

Cystic fibrosis-related diabetes (CFRD) is one of the most common extrapulmonary complications of CF. While CFRD only affects 2% of children, prevalence increases markedly with age, such that almost 20% of CF adolescents and 50% of CF adults will be diagnosed [49]. CFRD is associated with weight loss and reduced pulmonary status, and consequently an increase in morbidity and mortality [50]. Accordingly, there is great interest in the role of emerging CF therapies on the progression of CFRD. Despite the commonality and morbidity of CFRD, its pathogenesis has overlapping features shared by Type 1 diabetes mellitus (T1DM) and Type 2 DM. While the most well-understood cause of CFRD is decreased insulin secretion from the pancreas, it is likely a downstream consequence of defects in the CFTR protein encoded by mutations in the CF gene [51,52,53]. The CFTR protein is a chloride ion channel regulating the transport of chloride and bicarbonate in epithelial cells of many organs, the pancreas being one of them. In the presence of defective CFTR protein, thick secretions build up in the pancreatic ducts, leading to inflammatory and destructive injury to the pancreas and loss of insulin-producing beta cells. The lack of insulin is similar to that seen in T1DM, although the pathogenesis is not due to auto-antibodies as seen in T1DM [53,54]. Normal CFTR function is also responsible for beta-cell depolarization and release of insulin as well as alpha cell hyperpolarization and inhibition of glucagon release [51]. When CFTR is defective, there is dysregulation of beta and alpha cells, resulting in reduced insulin and increased glucagon release, respectively, and subsequently hyperglycemia. While HEMT has led to a significant improvement in the pulmonary and nutritional health of patients, there remains limited reports describing its role on glucose metabolism and its implications in the development and course of CFRD [52].

Large, randomized, controlled trials have yet to be seen reporting on the comprehensive impact of ETI on CFRD; such trials may not ever be feasible as HEMT is the current standard therapy for all eligible PwCF. However, a number of small studies have been published and add to our understanding of the role of ETI in CFRD. Korten et al. reported oral glucose tolerance test (OGTT) changes in 16 adolescents with CF 4–6 weeks after the initiation of ETI. Improvements at the 60-, 90- and 120-min intervals on OGTT were noted, indicating improvement in glucose tolerance, but no significant changes in insulin and c-peptide levels were found [55]. Similarly, in a study of 20 pediatric and adult CF patients followed for 10.5 months after initiation of ETI, there was no change identified in insulin resistance or insulin secretion, as measured by the homeostasis model assessment (HOMA-IR) and c-peptide index, respectively [56]. Unlike Korten et al., however, they did not find any significant changes in the 60- or 120-min interval on the OGTT; nor were any changes found in HbA1c or in the mean value of total glucose increases during OGTT post initiation of ETI [56]. In a single-center retrospective subgroup analysis of 134 patients with CFRD, no improvement in HbA1c was found over a mean of 12 months after the initiation of ETI [22]. Lastly, in a prospective, single-center, observational study reporting outcomes of continuous glucose monitor (CGM) measures after the initiation of ETI in adult CF patients, CGM measures of glucose control improved at a median of 7.1 months after ETI initiation [12]. In the larger-scale prospective, multi-center PROMISE study, preliminary data reported from this study did not show consistent improvements in glycemic outcomes, although this was limited to LUM/IVA and did not report on ETI [35].

While our understanding of the effects of ETI on insulin secretion and glycemic outcomes continues to evolve, it is important to also consider the nutrition guidelines that impact current diabetes care. Historically, high-calorie, high-fat diets have been recommended for all CF patients with malnutrition, including those with CFRD. However, modern CFRD medical nutrition guidelines prioritize good nutritional status while maintaining normal blood glucose values [57]. Recently, a prospective study of 18 adults with CF demonstrated a correlation between lower glycemic index foods and more stabilized glucose values [58]. Additionally, physical activity has been proven to lower post-prandial glucose values in adults with CF [59]. Although the benefits of ETI on glucose-control outcomes have yet to be fully understood, ETI has led to improvements in caloric intake, gastrointestinal function, and intestinal absorption, all of which are thought to contribute to weight gain and improvements in malnutrition [51]. Paradoxically, this has also led to an increased prevalence of obesity [51] and hypercholesterolemia in PwCF [60]. How these downstream effects of ETI will specifically affect patients with CFRD is yet to be seen; there have been limited studies focusing on nutrition and lifestyle management for CFRD since the introduction of ETI. There is a strong need to better understand the role of HEMT on CFRD; it is clear though that as HEMT continues to improve nutritional status in PwCF and as the population grows, CFRD dietary interventions will have to evolve as well, including education on quantity and quality of macronutrients (e.g., carbohydrates) alongside physical exercise recommendations [60,61].

### 3.4. Macronutrient Needs for Patients with CF and Advanced Liver Disease

The liver is commonly affected in those living with CF and is the third leading cause of death in PwCF. CF-related liver disease (CFLD) with cirrhosis and without cirrhosis is prevalent in 3.1% and 3.3%, respectively, of CF patients in the USA [62]. The long-term effect of CFTR modulators remain unclear and is under investigation in the PROMISE study, although there is evidence of benefit in the liver [35,61]. In a small study of 20 patients treated with LUM/IVA, there was a reduction in hepatic steatosis [63]. Treatment with ETI at an early age may even reduce or prevent severe CFLD. CFLD encompasses multiple liver-involved manifestations, such as neonatal cholestasis, elevated liver enzymes, steatosis, gallbladder and biliary tract disorders, cirrhosis, and portal hypertension. CFLD is classified into three categories: preclinical disease, CFLD without cirrhosis and portal hypertension (PH), and CFLD with cirrhosis and PH [64]. Treatment for CFLD includes treating the underlying cause for liver disease and monitoring nutritional status through the assessment of adequate caloric intake, PERT dosing, vitamin supplementation, particularly of the fat-soluble vitamins A, D, E, and K, and insulin regimens for those with CFRD. 

Patients with CFLD are at risk for malnutrition and micronutrient deficiencies due to decreased oral intake associated with anorexia, delayed gastric emptying, early satiety, increased energy expenditure, and malabsorption [65]. In CFLD, there is an estimated higher daily caloric intake of about 130–150% above general recommendations. Dietitians and CF clinicians must pay particular attention to patients’ intake of protein, fat, and carbohydrates. Patients with CFLD are more likely to need a higher intake of protein of 3 g/kg/day consumed in small, frequent daily portions to avoid stress on the liver [60]. Higher fat intake is also necessary to help meet increased energy demands in CFLD. In patients with cirrhosis, carbohydrates may not be properly utilized due to insulin resistance and glucose intolerance. They are at an increased risk of developing CFRD, and thus annual diabetes screening is important. Malnutrition is common in people with CFLD. Body mass index should not be the only indicator of nutritional status, rather the additional use of mid-arm circumference (MAC), triceps skinfold thickness, and hand grip should be used particularly in patients with ascites and/or edema. If patients cannot achieve adequate intake, enteral tube feeding such as nasogastric or gastrostomy tube can be used to maintain adequate nutritional status. 

Nutritional supplementation may be necessary to provide additional protein intake, along with the use of PERT to optimize fat absorption, absorption of micronutrients and fat-soluble vitamins to prevent deficiency. PERT dosing should be monitored annually to ensure absorption of essential fatty acids and nutrients, but it is important to be aware that malabsorption can happen in advanced CFLD that is unrelated to PERT dosing. Fat-soluble vitamins A, D, E, K, should be monitored every 6 to 12 months [66]. All vitamins should be taken with meals and enzymes if patients have PI. Vitamins K and A should be closely monitored in those with CFLD. Vitamin K deficiency is very common in CF although it is more common in those with CFLD. Prothrombin time should be monitored to access vitamin K stores indirectly. Patients with CFLD should receive an additional 5–10 mg of vitamin K daily, depending on the prolongation of prothrombin time [67]. In cirrhosis, retinol-binding protein is low because of the absence of hypoalbuminemia, causing low retinol levels. Patients with low retinol levels of less than 20 mcg/dL should receive vitamin A supplementation. It is important to monitor vitamin A levels closely since high levels of vitamin A can lead to hepatic toxicity. Salt supplementation should be avoided for those with cirrhosis and portal hypertension due to the risk of developing or worsening ascites.

### 3.5. Nutritional Influences in Kidney Stone Disease in CF

People with CF are at higher risk for kidney stone disease (KSD) or nephrolithiasis, with reported prevalence rates of 4.6% to 5.7%, over twice that of the general population. Active KSD screening in CF has yielded as high as 21% prevalence [68,69,70,71]. KSD may present urgently or emergently with acute pain and urinary symptoms requiring acute care visits or hospitalization. Nearly 38% of CF patients require at least one surgical intervention for KSD, compared to 10–20% seen in the general population [69]. Surgical management and general anesthesia also carry risks for respiratory complications, particularly in patients with advanced CF lung disease. Within one year, KSD prevalence of 11% was reported in one adult CF center with almost equal distribution between first-time diagnosis versus recurrent KSD [72]. Dietary queries focusing on KSD risk revealed high intake of sodium, animal protein, caffeine and sweetened beverages, and suboptimal intake of calcium, potassium and water consumption, all of which predispose to nephrolithiasis [72].

KSD risk factors associated with CF are well-described and can be modifiable for prevention; these include diet, metabolic predispositions, and fat malabsorption as a sequela of PI. Calcium oxalate stones are the most common type of kidney stone found in PwCF. Urinary abnormalities that predispose to stone formation in PwCF include low urine output (i.e., dehydration), hyperoxaluria, hypocitraturia, and hypercalciuria [73,74]. PwCF are frequently prescribed antibiotics for pulmonary exacerbations; over time, frequent gut exposure to antibiotics can alter the GI flora. Antibiotics also decrease the breakdown of oxalate, causing hyperoxaluria. Reported mean age of onset for KSD in CF is early adulthood however stone recurrence is as high as 43% [69,73]. The CF community is seeing new emerging complexities in CF care in the era of HEMT and it is key that the CF care team has an increased awareness of new illnesses not commonly seen in PwCF in the past. Increasing age of PwCF may be associated with increasing prevalence of KSD, and obesity may be on the rise as well. This highlights the need to address dietary preventative measures to reduce risk for KSD and its associated healthcare burden.

## 4. Nutritional CF Care in Unique Conditions

### 4.1. Nutrition in Pregnancy with Cystic Fibrosis 

Advances in HEMT have led to an increased rate of pregnancies in PwCF, believed to be related to an improvement in cervical mucous viscosity and overall improved health and life expectancy. The CF community is just beginning to learn more about pregnancy courses in women with CF following the approval of HEMT. The MAYFLOWERS trial is following maternal and fetal outcomes in pregnant CF patients with the objective of monitoring lung function in women with CF during their pregnancy and two years postpartum while being on HEMT [75]. At this time, there are no existing clinical care guidelines specific to pregnancy in CF including nutritional care; thus, we refer to general guidelines related to nutritional health in pregnancy and make note of any unique circumstances relevant to CF. 

Nutritional status during pregnancy is monitored using BMI, micronutrient and macronutrient levels, evaluating food intake through recall diaries for example, and completing a nutrient analysis by calculating daily intake of protein, carbohydrates, fats, sugars, fats, sodium and fiber, and routine follow ups with a registered dietitian. General weight gain recommendations are shown in Table 3 in order to maintain a healthy pregnancy and maternal and fetal weight gain. Impaired pre-pregnancy nutrition is a significant risk factor for suboptimal maternal and fetal outcomes, such as newborns with low birth weight, intrauterine growth restriction and reduced maternal nutrition supply to the fetus [76,77]. Women having a normal preconception bodyweight or BMI (18.5 to 24.9), have been associated with more favorable pregnancy outcomes, and reduced risk of requiring a caesarean section. When patients are underweight, there is an imbalance of hormones in the body, which causes the body to make less estrogen, and many underweight women cease to have periods due to the lack of estrogen [78].

Nutritional screening as performed in general CF care should continue as it relates to assessment of micronutrient status, which is best carried out before conception. This requires an assessment of dietary intake and blood levels. PI leads to malabsorption of fat-soluble vitamins A, D, E, and K and requires supplementation. Goal dosing and fetal development relevance of vitamins and minerals in pregnancy are summarized in Table 4. Vitamin A supplements in deficient pregnant women reduces the incidence of maternal anemia and is associated with a reduced incidence of maternal infections. Vitamin D deficiency is associated with increased risk of gestational diabetes and preeclampsia. Vitamin E supplements should be adjusted based on blood levels, and vitamin K should be continued if needed prior to pregnancy. Continuous folic acid supplementation for at least 12 weeks and regular follow up with a CF dietitian is recommended [79]. Prenatal care will vary depending on the patient’s symptoms and severity of their pregnancy. Patients are encouraged to follow up with their multidisciplinary CF team and obstetrician. 

### 4.2. Nutrition & Lung Transplantation

Following the approval of ETI, rates of lung transplantation (LTx) for CF have declined, although it remains a treatment option in patients with advanced CF lung disease, particularly if there is worsening nutritional status despite supplementation [80]. According to CFF consensus guidelines, all adults with CF should be referred when (1) FEV_1_ is <30% predicted; (2) FEV_1_ < 40% predicted accompanied by at least two pulmonary exacerbations and/or massive hemoptysis; (3) FEV1 is <50% predicted with a rapid decline of more than 20% FEV1 over one year, and/or sooner if other severity indicators are present, including a BMI < 18, and signs of advanced lung disease such as oxygen dependency, hypercapnia, and/or pulmonary hypertension [81,82]. 

Optimization of nutritional status before LTx is vital and a key reason why early referral is recommended, to allow for adequate time to address any malnutrition concerns. Malnutrition is a modifiable risk factor and a widespread problem in PwCF for all the reasons previously mentioned: increased metabolic demands associated with recurrent infections, chronic inflammation, and increased respiratory effort. Pre-LTx nutritional management includes adherence to a high-calorie/high-fat diet, PERT, vitamin and mineral replacement, calorie dense oral nutritional supplements, and enteral support if needed [83]. Lean body mass or fat-free muscle mass correlates with inspiratory muscle function [84]. This suggests that preserving fat-free mass stores may play an important role in diaphragmatic function in advanced lung disease, including in patients awaiting LTx. Depletion of fat-free mass is strongly associated with increased mortality while awaiting LTx and prolonged post-transplant intensive care unit stays [85]. While low BMI of <17 kg/m^2^ or <18 kg/m^2^ is commonly a contraindication for LTx for all lung diseases in many transplant centers, outcomes in CF transplant recipients with such low BMI may fare better than expected. In a study using the United Network for Organ Sharing Registry data between 2005–2015, post-transplant mortality among PwCF demonstrated a reasonable median post-transplant survival of 7 years in CF patients with pre-transplant BMI <17 kg/m^2^ which is comparable to other advanced lung disease groups [81]. 

On the other end of the spectrum are patients with advanced lung disease and obesity; approximately one third of PwCF are overweight or obese [86]. A BMI >35 kg/m^2^ is considered a relative contraindication to LTx by the International Society for Heart and Lung Transplantation as there is evidence of an increased risk of primary graft dysfunction and posttransplant mortality for obese recipients compared to normal or overweight candidates [87]. The prevalence of being overweight and obesity in the CF population follows the introduction of HEMT use. CF care teams and specifically dieticians will need to remain vigilant in nutritional education and diet modifications tailored to patients’ overall health. Predictors of posttransplant outcomes in CF lung recipients as they relate to nutritional status will surely need to be reevaluated in the era of HEMT and may influence the existing criteria thresholds for referral and listing [80].

## 5. Conclusions

People with CF are living longer, healthier lives following the approval of HEMT. The long-term effects of HEMT on nutritional status have yet to be established; more research is needed and further understanding will likely shape standard of care and clinical practices. The goal of current dietary recommendations for PwCF is to deliver more than 100% of estimated energy needs through a high-fat diet; however, studies are now showing reduced energy expenditure in the setting of HEMT use. Dietary modifications will likely be more variable going forward as some patients who are ineligible for HEMT or remain malnourished/underweight despite HEMT will continue to need the traditional CF diet on one side of the spectrum, while patients becoming overweight or obese (more likely on HEMT) will need less caloric diets on the opposite end of the spectrum. The traditional high-calorie, high-fat, salt-supplemented diet also poses a risk for cardio-metabolic disease in the general population and may in time become more apparent in the aging CF population [87,88]. In essence, guidelines will need to be adjusted on an individual basis. The evolution of the nutritional landscape in CF will be followed closely by the CF community.

## Figures and Tables

**Table 3:** General weight-gain recommendations for pregnancy.

Pre-Pregnancy Weight Category	Body Mass Index	Recommended Range of Total Weight Gain throughout Pregnancy
Underweight	Less than 18.5	28–40
Normal weight	18.5–24.9	25–35
Overweight	25–29.9	15–25
Obese (all classes)	30 or greater	11–20

**Table 4:** Pregnancy vitamin and minerals recommendations.

Vitamins and Minerals	Fetal Development
Folic Acid (400–600 mcg/daily)	Supports the placenta and prevents spina bifida and other neural tube defects
Protein (75–100 g/daily)	Produces amino acids, and repairs cell
Iron (27 mg/daily)	Helps with hemoglobin production, prevents anemia
Calcium (1000 mg/daily)	Creates healthy bones, prevents blood clots
Vitamin A/ Beta Carotene (770 mcg, daily max 1000 mcg)	Promotes bone and teeth growth
Vitamin D (600 IU/daily)	Helps body use calcium and phosphorus to promote strong bones
Vitamin E (15 mg/daily)	Forms red blood cells and muscles
Vitamin C (85 mg/daily)	Protect against tissue damage, helps absorb iron
Docosahexaenoic acid—DHA (200 mg/daily)	Helps with brain development

## Data Availability

Not applicable. No new data was created.
